# Invasive Alien Plants in Africa and the Potential Emergence of Mosquito-Borne Arboviral Diseases—A Review and Research Outlook

**DOI:** 10.3390/v13010032

**Published:** 2020-12-27

**Authors:** Sheila B. Agha, Miguel Alvarez, Mathias Becker, Eric M. Fèvre, Sandra Junglen, Christian Borgemeister

**Affiliations:** 1 Centre for Development Research (ZEF), University of Bonn, Genscheralle 3, 53113 Bonn, Germany; cb@uni-bonn.de; 2 International Livestock Research Institute, Old Naivasha Road, P.O. Box 30709, Nairobi 00100, Kenya; Eric.Fevre@liverpool.ac.uk; 3 Institute of Crop Science and Resource Conservation (INRES), Department of Plant Nutrition, University of Bonn, Karlrobert-Kreiten-Strasse 13, 53115 Bonn, Germany; malvarez@uni-bonn.de (M.A.); mathias.becker@uni-bonn.de (M.B.); 4 Institute of Infection, Veterinary and Ecological Sciences, University of Liverpool, Leahurst Campus, Chester High Road, Neston CH64 7TE, UK; 5 Institute of Virology, Charité-Universitätsmedizin Berlin, Corporate Member of Free University Berlin, Humboldt-University Berlin, and Berlin Institute of Health, 10117 Berlin, Germany; sandra.junglen@charite.de

**Keywords:** agricultural intensification, agricultural expansion, arboviral disease vectors, invasive plants, *Lantana camara*, land-use changes, mosquito ecology, *Opuntia ficus-indica*, pathogen transmission, *Prosopis juliflora*, *Parthenium hysterophorus*

## Abstract

The emergence of arthropod-borne viruses (arboviruses) as linked to land-use changes, especially the growing agricultural intensification and expansion efforts in rural parts of Africa, is of growing health concern. This places an additional burden on health systems as drugs, vaccines, and effective vector-control measures against arboviruses and their vectors remain lacking. An integrated One Health approach holds potential in the control and prevention of arboviruses. Land-use changes favour invasion by invasive alien plants (IAPs) and investigating their impact on mosquito populations may offer a new dimension to our understanding of arbovirus emergence. Of prime importance to understand is how IAPs influence mosquito life-history traits and how this may affect transmission of arboviruses to mammalian hosts, questions that we are exploring in this review. Potential effects of IAPs may be significant, including supporting the proliferation of immature and adult stages of mosquito vectors, providing additional nutrition and suitable microhabitats, and a possible interaction between ingested secondary plant metabolites and arboviruses. We conclude that aspects of vector biology are differentially affected by individual IAPs and that while some plants may have the potential to indirectly increase the risk of transmission of certain arboviruses by their direct interaction with the vectors, the reverse holds for other IAPs. In addition, we highlight priority research areas to improve our understanding of the potential health impacts of IAPs.

## 1. Introduction

Large-scale land-use changes, especially as a result of a drive towards agricultural intensification and expansion, are progressively re-shaping the future of rural Africa. The prime driver of this change remains the need to improve food security given the high population growth on the continent, leading to an estimated doubling of the population by 2050 [1]. Agricultural practices in Africa may have unintended health implications by driving the emergence of arthropod borne diseases [2]. Many important zoonotic diseases caused by arthropod-borne viruses (arboviruses) like Rift valley fever (RVF), West Nile, dengue, yellow fever, chikungunya, and Zika originated in Africa, and their initial emergence might have remained unnoticed as human encroachment into natural ecosystems, deforestation, and agricultural transformation was taking place. Yet, the arthropod vectors that transmit these arboviruses are particularly sensitive to land-use changes [3,4,5] and can quickly adapt to changing ecosystems by altering key aspects of their bionomics.

The emergence of previously unknown viruses and the re-emergence of known ones are a huge challenge to both the human and veterinary health sectors as the outbreaks they cause remain hard to predict and difficult to combat. Though an integrated One Health approach, which combines human, animal, and environmental health, could contribute in controlling arboviral diseases and in curbing their emergence and spread, these efforts are presently hampered by a lack of knowledge on the functional environmental linkages that can serve as a proxy to predicting arbovirus emergence. Moreover, certain aspects of the biology and ecology of arboviral vectors remain poorly understood, especially in the case of mosquitoes; chief amongst the less appreciated aspects of their biology is the role of plants in the feeding and survival of female members of the population. Though both male and female mosquitoes repeatedly ingest sugars available in plants to sustain daily metabolic activities, thus ensuring survival and flight [6,7], evidence increasingly suggests that female mosquitoes also depend on certain plants in the environment for different aspects of their bionomics. This may be for (i) enhancing their reproductive success [8,9], (ii) use as microhabitats [10], (iii) use for the proliferation of larvae [11,12], and (iv) for blocking transmission of pathogens [13]. These aspects of mosquito biology would potentially be affected by changes in the vegetation structure and composition. Strong differences of mosquito abundance [14,15] and survival [8,9,16] have been observed between habitats colonized by nectar-rich and nectar-poor plants. Whether invasive alien plants (IAPs) might affect the risk of emergence of malaria was recently reviewed by Stone et al. [7], but little is known on possible linkages between IAPs and life-history traits of arboviral vectors, in particular of mosquito vectors. Lately some IAPs are reported to affect the health of humans and livestock, thus adding to their already considerable economic and environmental effects [17,18,19].

In this review, we explore how vector bionomics and competence (i.e., the ability to transmit viruses) are affected by IAPs on the African environment. We focus on mosquitoes because these are the principal vectors of most arboviruses, and their biology and ecology are best understood. However, because of the limited existing knowledge on arboviral mosquito vectors we also refer to data from malaria and its mosquito vectors and explore how this knowledge can inform future research on arboviral diseases. The latter forms the second major objective of this review as we seek to identify areas where more research efforts are needed to develop a framework for future research in arbovirology. Critical evaluations will be made on individual IAPs and mosquito vector species, along the life-history of the latter, and where possible information will be provided on the inhibiting potential of IAPs against arboviruses. Exploring the link between IAPs and the emergence of arboviral diseases may form the basis for recommending effective control measures and contribute to an integrated approach to arboviral diseases management.

## 2. Invasive Alien Plants

Invasive plant species are defined by three main properties, (i) their bio-geographical origin (alien plants, introduced either accidentally or on purpose by living organisms), (ii) their ability to spread without human assistance (aggressive invasion of pristine environments or opportunistic establishment in disturbed habitats), and (iii) their negative effects, including economic, environmental, and aesthetic impacts [20]. Most species of IAPs belong to the three plant families Compositae, Poaceae, and Leguminosae [21]. The IAPs reviewed here are among those with potential relevance to arthropods and particularly to virus-transmitting mosquitoes. In particular, these are species that are able to rapidly fill “open” niches by fast growth and aggressive spread dynamics [22], and that occupy ecological niches similar to those preferred by disease vectors [23]. This concerns open water surfaces (IAPs colonizing aquatic and semi-aquatic habitats) or anthropogenically disturbed terrestrial areas where IAPs displace natural vegetation using allelochemicals [24] and by effectively competing for resources such as light, water, and nutrients [25]. In these cases, IAPs may substantially alter ecological conditions, differentially affecting arboviral mosquito vectors at different stages of their development. The main group of plants discussed in this review correspond to (i) floating aquatic plants and semi-aquatic helophytes, (ii) annual and perennial forbs, (iii) woody species, and (iv) succulent plants (Table 1). 

Aquatic and semi-aquatic environments have been greatly affected by IAPs in Africa. Floating species such as water hyacinth (*Eichhornia crassipes*), water lettuce (*Pistia stratiotes*), and water fern (*Salvinia molesta*) are considered among the earliest introductions of alien plants into Africa [26,27,28]. Due to the azonality of aquatic environments, most of those species are ubiquitous. The neotropical species *Mimosa pigra* are predominant in semi-aquatic habitats such as swamps and floodplains. These aquatic IAPs were introduced into Africa as ornamental plants for artificial ponds and are causing huge ecological and economic problems [19]. 

Annual and perennial herbs mainly colonize ruderal (disturbed) places such as roadsides, quarries, and construction places [29], but may also occur as common weeds (segetal flora) in irrigated and rain-fed croplands [30]. Examples of IAPs in cultivated and fallow lands are the annuals *Argemone mexicana*, *Bidens pilosa*, *Datura stramonium*, *Galinsoga parviflora*, *Tagetes minuta,* and *Parthenium hysterophorus*. Because these weeds are short-lived and often outcompeted by perennial forbs and woods, their effects on the environment are usually neglected. However, of emerging ecological concern is *P. hysterophorus* due to its fast spread and dominant growth [29,31,32]. 

Woody species (trees and shrubs) are the most important group of IAPs based on their size and impact on vegetation structure. Many are broadly distributed across the continent in tropical and sub-tropical areas like the shrubs *Chromolaena odorata* [33,34] and *Lantana camara* [35,36]. The latter is often grown as a live fence around homes and occasionally croplands and is a preferred habitat of tsetse flies (*Glossina* spp.), the vectors carrying the parasitic protozoan that causes trypanosomiasis in humans and animals [37]. Others can invade protected areas like *Broussonetia papyrifera* [38], *Psidium guajava* [39], and *Senna spectabilis* [40]. Of greatest concern in this respect in sub-Saharan Africa and beyond is the rapid spread of *Prosopis juliflora* (mesquite), considered as one of the world’s worst invasive plant across the tropics [41,42]. 

Succulent plants are adapted to semi-arid conditions, occurring in stony shallow soils that are frequently used for rain-fed cultivation or as seasonal grazing places. Succulent IAPs, often neotropical Cactaceae like *Austrocylindropuntia subulata*, *Opuntia ficus-indica,* and *O. stricta*, frequently outcompete native succulents [19,43]. 

## 3. Effect of Invasive Alien Plants on the Ecology of Preimaginal Stages of Arboviral Mosquito Vectors

Lentic and temporal aquatic habitats are essential for mosquito development, supporting the growth and survival of immature stages of the vectors [44,45]. Though the immature stages of arboviral mosquito vectors may develop in aquatic habitats devoid of plants, like in *Aedes aegypti* [44], most arboviral mosquito vectors including *Culex*, *Mansonia*, and other *Aedes* species preferentially develop in or are attracted to water habitats containing plants or plant debris [45]. 

**Figure 1:**
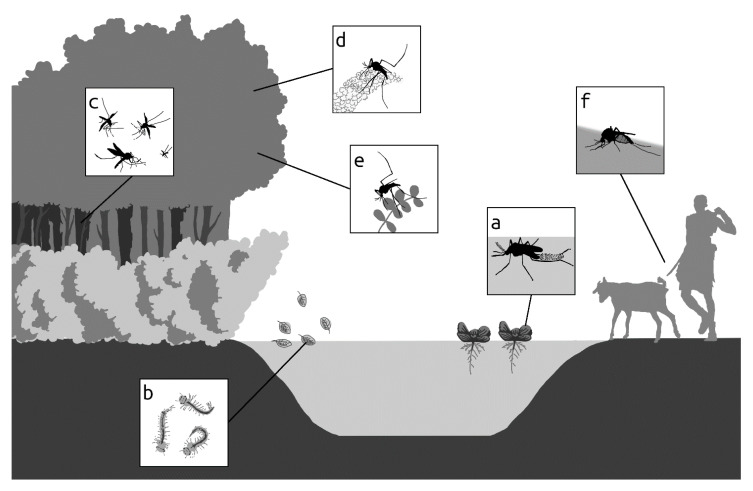
Potential effects of invasive plants on mosquito life-history traits. (**a**) Invasive plants offer suitable oviposition sites for some vector species; (**b**) invasive plant litter increases proliferation of immature vectors; (**c**) dense canopy cover or thickets of invasive plants provide suitable micro-habitats for adult mosquitoes; (**d**) nectariferous flowers (**e**) and extra-floral glands of invasive plants are important sugar sources for adult vectors; (**f**) invasive plants can influence the pathogen transmission ability of the vector. (designed by Miguel Alvarez).

Some aquatic plants are known to support mosquito breeding [46,47] (Figure 1a). For instance, the invasive water fern *S. molesta* has been associated with higher egg laying capacity of *Cx. annulirostris* [47], a potential vector of Rift Valley fever virus (RVFV) [48,49] and West Nile virus (WNV) [50]. In addition, the roots of the invasive water lettuce *P. stratiotes* are a favourable breeding site for *Mansonia* spp., a secondary RVFV vector [51], indicating the potential indirect impact that the control of these IAPs could have on arboviral diseases in humans and livestock. Besides aquatic plants, the presence of debris (from leaves, fruits, seeds, flowers, pollen) from terrestrial plants growing in the neighbourhood of temporal water bodies has the potential to alter the chemistry of such environments. This may influence their attractiveness as breeding sites for gravid female mosquitoes, with inherent consequences for the resulting larval development and adult emergence rates [12,52,53] (Figure 1b). 

In addition to the indirect facilitation of the development of Cyanobacteria by floating aquatic plants [54], the presence of plant debris or inorganic material may increase the Nitrogen (N) and Phosphorus (P) content of the water, thereby supporting the growth of micro-organisms, possibly leading to increased mosquito oviposition, since the emerging larvae depend on Cyanobacteria as food for their development [55,56]. This is particularly true for *Cx. pipiens*, the primary vector of WNV and secondary vector of RVFV, which is known to thrive in habitats that contain high amounts of organic material, often as a result of human and livestock activities [57]. Decaying *L. camara* leaves for instance, have high N and P release rates [58], and the presence of its leaf litter has been shown to significantly drive increases in immature *Culex* spp. and *Aedes* spp. compared to those of the native plant *Terminalia sericea* [11]. However, despite the extremely progressive invasion history of Prosopis in semi-arid regions of Africa [25,42,59], little is known on the potential effects of Prosopis debris on the development of immature arboviral mosquito vectors in comparison to local native plants. Thus, this needs to be investigated. 

Furthermore, the thickets formed by these IAPs may provide a suitable moist and humid microhabitat conducive for egg laying as well as some protection against desiccation, especially during lengthy periods of drought. This will particularly be beneficial for floodwater *Aedes* spp. (primary and maintenance vectors for the RVFV), that need to remain viable during extended periods of drought. Elucidating the potential role IAPs play in supporting the development of immature stages of important arboviral mosquito vectors could greatly improve our understanding of the distribution of these vectors as well as the dynamics of the diseases they transmit. Future research should address, among others, whether (i) IAP debris can alter the oviposition activities of certain mosquito vectors, thus affecting larval and subsequently adult emergence, and (ii) if the development of immature mosquitoes in IAP debris infested breeding sites affects the ability of the emerging vectors to transmit arboviruses. Such data are important for identifying productive breeding sites for targeted vector and/or IAPs control, as well as for identifying hot spot areas for arbovirus emergence. 

## 4. Effect of Invasive Alien Plants on Arboviral Mosquito Vector Survival

Vector density and distribution are important determinants for the distribution pattern of arboviral diseases. This can be driven, among other factors, by the availability of water and suitable resting sites or shelters (Figure 1c), and the presence of plants for sugar-feeding (Figure 1d,e). Interestingly, urban areas with moderate to high terrestrial vegetation covers were associated with higher abundance of WNV vectors *Cx. pipiens*, *Cx. restuans*, *Cx. salinarius,* and *Ae. vexans* [60]. Green vegetation has also been identified as an important risk factor in RVF outbreaks [61,62,63]. Though the specific plant species in the vegetation driving the observed patterns in these studies remain unknown, it is obvious that vegetation plays an essential part in ensuring vector survival or longevity, thus the need to critically evaluate the potential contributory role of individual plant species in the environment. Many IAPs flower longer and are thus more readily available sources for vector plant-sugar feeding. Furthermore, arthropod vectors appeared to preferentially obtain sugar from invasive plants compared to native plants in a cage experiment [64]. This makes it even more important to establish a comparative research analysis on the effect of invasive and native plants on arboviral vectors bionomics. 

Similarly, the impenetrable thickets formed by woody IAPs like *P. juliflora* can provide favourable microhabitats with access to water and shelter for mosquito vectors, especially in semi-arid regions of Africa, therefore ensuring diurnal survival. Thus, it is quite likely that the presence of certain IAPs in an environment impact vector survival and consequently their abundance and distribution [14,15]. The potential implications of *P. juliflora* on the health of local communities, through favouring the development of arboviral mosquito vectors, adds a new dimension to the already ambiguous effects this IAP can have on community livelihoods through provision of fuel wood on one hand and the invasion of rangeland on the other [65].

Though there is currently no evidence for a direct link between the presence of IAPs to an increased abundance of arboviral mosquito vectors in east Africa, higher malaria incidence in Prosopis invaded areas around Lake Baringo was associated with Prosopis invasion based on perceptions by the community [65]. Furthermore, a habitat manipulation experiment in Mali showed that eliminating flowering branches from Prosopis plants lead to a drop in the malaria vector population by 69%, thereby confirming the perceptions of the local community [15]. *Prosopis juliflora* has nectariferous flowers and an extremely precocious flowering age (3 to 5 years after seedling establishment) [66], which may be an important source of nutrition for vectors. Furthermore, *P. juliflora* as well as many other Leguminosae (e.g., *Acacia* spp. and *Senna* spp.) have extra-floral nectar glands situated on the leaves [67] (Figure 1e), which may offer nectar to arboviral mosquito vectors for longer periods than flowers. Additionally, in a cage experiment, *Ae. aegypti* mosquitoes were reported to feed (93%) and survive best (86%) on flowing *P. juliflora* compared to most other plants [68]. Flowering *P. juliflora* were additionally considered to be significantly more attractive to *Ae. aegypti* [68]. These preliminary findings emphasise the need for more conclusive research on the role of *P. juliflora* on vectors of arboviral diseases. 

*Parthenium hysterophorus* has the ability to suppress the growth of vegetation in a wide range of ecosystems, which has enabled its vast spread in Africa. Its wide occurrence around homes, crop-, and rangelands, partly as a result of agricultural intensification measures like irrigation, may favour plant feeding by mosquitoes. In fact, the malaria vector *An. gambiae* preferentially feeds on *P. hysterophorus*, but not because of its nutritional value, which turned out to be rather inferior for *An. gambiae* [8,9,69], but possibly because of its attractive odour [70] or the anti-plasmodium properties of some of its secondary plant metabolites [13] (further discussed in the next section). Thus, plant-feeding on *P. hysterophorus* could potentially suppress vector populations over time through negative effects on the fitness of the females [8,9,69]. Yet, Nyasembe et al. [71] found that *An. gambiae* after feeding on *P. hysterophorus* actually survived better and hypothesized that this could negatively impact malaria control in Africa. In addition, higher human biting rates were recorded among malaria vectors exposed to sugar-poor IAPs like *P. hysterophorus* [16], thereby increasing the risk of vector-borne disease spread. While it is important to resolve the ambiguity around *P. hysterophorus* in terms of its potential effect on the risk of malaria transmission, it is worth noting that research on *P. hysterophorus* has so far been limited to *An. gambiae*. To investigate if *P. hysterophorus* will have a similar effect on arboviral vectors, it will be important to conduct studies to show if arboviral vectors feed on this plant, and if so, then followed by an assessment of the effect on vector survival and reproductive fitness.

Similarly ambiguous are the effects of *L. camara* on arboviral mosquito vectors. Though there are some indications that *L. camara* leaf litter can favour development of larvae of arboviral mosquito vectors [11], *L. camara* has relatively long corollas [64] and despite the high sugar content in the flowers, its floral architecture makes plant feeding rather difficult. In a detailed study involving *An. gambiae* and several plant species *L. camara* ranked among the least preferred nectar sources and mosquitoes that had fed on it subsequently demonstrated lower fecundity and a shorter life span [9,64]. Moreover, survival rates on *L. camara* were no better than on water, most likely because of the low sugar content of *L. camara* leaves (where the vector turns to feed) [72]. However, as in the case of Prosopis, thickets formed by clusters of *L. camara* might provide a favourable microhabitat for adult arboviral mosquito vectors as reported for tsetse flies [37,73]. Trying to resolve this ambiguity around *L. camara* should be another focus of future research. 

*Opuntia ficus-indica* was widely introduced throughout the world as a commercial fruit, fodder crop, ornamental, and “natural fence” [19]. Mature fruits from *O. ficus-indica* contain high amounts of reducing sugars (glucose and fructose), and field caught *Ae. mcintoshi* and *Ae. ochraceus*, the primary RVFV vectors, have been reported to feed on the plant [74]. Yet, how and to what extend this might affect mosquito survival remains to be investigated. Like many other IAPs, *O. ficus-indica* and the congener *O. stricta* invade protected, disturbed, or abandoned agricultural lands and eventually replace native vegetations [19,43]. As it also forms impenetrable thickets, *O. ficus-indica* might provide arboviral mosquito vectors with favourable habitats in otherwise inhospitable semi-arid environments. Yet, apart from initial observations by Nyasembe et al. ([74] little is known on the relationship between *O. ficus-indica* and arboviral mosquito vectors. We hypothesize that *Ae. mcintosh* and *Ae. ochraceus,* sugar-feeding on *Opuntia* spp., offer these RVFV vectors a competitive advantage in their survival and reproductive fitness compared to naturally occurring plants. However, experimental survival analyses to support this hypothesis are so far lacking.

For many important IAPs in Africa, nothing is known on the effects that these plants have on the physiology of arboviral mosquito vectors. Studying the putative influence of IAPs on mosquito vectors could contribute to the development of a holistic integrated vector control effort. 

## 5. Potential Effects of Invasive Alien Plants on Viral Pathogen Transmission

Mosquito species of the *Aedes, Culex, Mansonia, Eretmapodites*, and *Anopheles* genera are tremendous threats to public health due to their ability to transmit viruses to humans and/or livestock [75]. Because viral susceptibility and transmission by a mosquito vector is affected by nutrition [76], the choice of nutrition is critical for the propagation of infectious pathogens to humans and livestock (Figure 1f). However, little to nothing is known about the role of plant nutrition of the mosquito vector on viral infection, replication, and transmission. 

Besides the oviposition altering properties of plant debris in vector breeding sites, histopathological evidence showed that consumption of decaying plant leaves by larvae of *Ae. aegypti, Ae. albopictus*, and *Cx. pipiens* affected their midgut epithelial cells [77]. Disruption of the midgut epithelial cells may interfere with immature development, adult emergence, and possibly pathogen transmission abilities [78]. Though this does not directly point to all IAPs, it is known that the aquatic IAP *Eichhornia crassipes* is able to affect the midgut epithelia tissue of *Cx. pipiens* [79]. In addition, the fact that leaf litter of certain IAPs enhances the abundance of *Culex* spp. and *Aedes* spp. [11], makes it particularly interesting to investigate whether these plants also affect the adult vectors’ fitness and susceptibility to viral pathogens. 

Both male and female mosquitoes rely on plant sugar for flight and survival [6,7], with possible additional reproductive and pathogen susceptibility benefits for female mosquitoes [9,13]. Recent studies have confirmed that certain secondary metabolites (toxins) from plants are ingested by mosquito vectors along with common plant sugars [80]. However, the fate and impact of the ingested plant secondary metabolites remains unclear, particularly for arboviral mosquito vectors. For malaria it is known that *An. gambiae* preferentially feeds on *P. hysterophorus*, though because of the plant’s relatively inferior sugar profile it appears that in terms of nutrition the plant does not have any adaptive significance to the vector [8,9,64,69]. Hence the vector’s feeding preference for *P. hysterophorus* could be a mere reflection of its wide availability caused by the rapid replacement of native vegetation by this highly aggressive IAP, its attractive odour [70], or due to other underlying physiological benefits that the plant offers the vector. It was therefore hypothesized that besides sugars, other constituents present in *P. hysterophorus* could benefit *An. gambiae* [64]. Parthenin, a sesquiterpene lactone present in *P. hysterophorus,* was isolated from recently plant-fed *An. gambiae* [80] and was later shown to block the transmission of the asexual stages of *Plasmodium falciparum* in infected mosquitoes [13], leading to speculations that the female mosquitoes preferentially seek sugar from *P. hysterophorus* to rid themselves of a plasmodium infection [80]. Though some of the anti-microbial properties of parthenin are well documented [13,81], its antiviral potential remains unknown and merits further investigation. 

Thus, based on the recent promising results regarding malaria vectors [13,80], the potential suppressing or even enhancing properties of secondary plant metabolites on arbovirus vectors, especially those found in aggressively spreading IAPs in Africa, demands greater scientific attention. This should be accompanied by more detailed studies on plant feeding preferences in arboviral mosquito vectors, including the dengue vector *Ae. aegypti* but also vectors of other important arboviral diseases like RVFV. 

## 6. Conclusions

The plant assemblage in an environment has the potential to influence the abundance and diversity of mosquito vectors and their ability to transmit infectious pathogens. In Africa, IAPs are dramatically altering ecologies and whole landscapes. We believe that this has profound consequences on the dynamics of arboviral mosquito vectors and on the epidemiology of important endemic arboviral diseases on the continent. Although not much is presently known, some initial studies on arboviral vectors and IAPs (immature development, plant sugar feeding preference, survival, reproductive fitness, and vector competence) are indicating a whole new area of research, which eventually might enrich our portfolio of integrated control options and risk analysis mapping of vector-borne viral diseases. 

## Figures and Tables

**Table 1:** Vascular plant species cited in this article as invasive for African countries and grouped according to their life forms.

Scientific Name	Common English Name	Family	Geographical Origin
A. Aquatic and semi-aquatic plants			
*Argemone mexicana* L.	Mexican poppy	Papaveraceae	Central America and the Caribbean
*Eichhornia crassipes* (Mart.) Solms	Water hyacinth	Pontederiaceae	Tropical America
*Mimosa pigra* L.	Giant sensitive plant	Leguminosae	Tropical America
*Pistia stratiotes* L.	Water lettuce	Araceae	Probably Tropical America
*Salvinia molesta* D. S. Mitch.	Giant salvinia	Salviniaceae	Tropical America
B. Annual herbs and ruderal forbs			
*Bidens pilosa* L.	Blackjack	Compositae	Tropical America
*Datura stramonium* L.	Thorn apple	Solanaceae	North America
*Galinsoga parviflora* Cav.	Quickweed	Compositae	Central America and the Caribbean
*Parthenium hysterophorus* L.	Famine weed	Compositae	Tropical America
*Tagetes minuta* L.	Wild marigold	Compositae	South America
C. Shrubs and trees			
*Broussonetia papyrifera* (L.) L’Hér. ex Vent.	Paper mulberry	Moraceae	Subtropical Asia
*Chromolaena odorata* (L.) R. M. King & H. Rob.	Siam weed	Compositae	Tropical America
*Lantana camara* L.	Lantana	Verbenaceae	Tropical America
*Prosopis juliflora* (Sw.) DC.	Mesquite	Leguminosae	Tropical America
*Psidium guajava* L.	Common guava	Myrtaceae	Central America and the Caribbean
*Senna spectabilis* (DC.) H.S. Irwin & Barneby	Golden wonder tree	Leguminosae	Tropical America
D. Succulent plants			
*Austrocylindropuntia subulata* (Muehlenpf.) Backeb.	Long-spine cactus	Cactaceae	Peruvian Andes
*Opuntia ficus-indica* (L.) Mill.	Sweet prickly pear	Cactaceae	North America
*Opuntia stricta* (Haw.) Haw.	Erect prickly pear	Cactaceae	North America

## Data Availability

Data is contained within the article.
